# Transitioning Focus Group Research to a Videoconferencing Environment: A Descriptive Analysis of Interactivity

**DOI:** 10.3390/pharmacy9030117

**Published:** 2021-06-24

**Authors:** Cristine B. Henage, Stefanie P. Ferreri, Courtney Schlusser, Tamera D. Hughes, Lori T. Armistead, Casey J. Kelley, Joshua D. Niznik, Jan Busby-Whitehead, Ellen Roberts

**Affiliations:** 1 Center for Aging and Health, Division of Geriatric Medicine, School of Medicine, The University of North Carolina at Chapel Hill, Chapel Hill, NC 27599, USA; cjkelley@med.unc.edu (C.J.K.); jdniznik@email.unc.edu (J.D.N.); jan_busby-whitehead@med.unc.edu (J.B.-W.); ellen_roberts@med.unc.edu (E.R.); 2 Division of Practice Advancement and Clinical Education, Eshelman School of Pharmacy, The University of North Carolina at Chapel Hill, Chapel Hill, NC 27599, USA; Stefanie_Ferreri@unc.edu (S.P.F.); tdhughes@email.unc.edu (T.D.H.); lori_armistead@unc.edu (L.T.A.); 3 Department of Epidemiology, Gillings School of Global Public Health, The University of North Carolina at Chapel Hill, Chapel Hill, NC 27599, USA; courtney.schlusser@unc.edu; 4 Division of Pharmaceutical Outcomes and Policy, Eshelman School of Pharmacy, The University of North Carolina at Chapel Hill, Chapel Hill, NC 27599, USA

**Keywords:** focus groups, videoconferencing, deprescribing, telecommunication

## Abstract

The COVID-19 pandemic disrupted face-to-face interactions in healthcare research, with many studies shifting to video-based data collection for qualitative research. This study describes the interactivity achieved in a videoconferencing focus group of seven primary care providers discussing deprescribing opioids and benzodiazepines. Researchers reviewed video footage of a focus group conducted via Zoom and assessed interactivity using Morgan’s framework for focus group communication processes. Two reviewers categorized the type of exchanges as sharing information, comparing experiences, organizing, and conceptualizing the content, as well as validating each other or galvanizing the discussion with “lightning strike” ideas. The conversation dynamics in this focus group included clear examples of interactivity in each of the categories proposed by Morgan (validating, sharing, comparing, organizing, conceptualizing, and lightning strikes) that were observed by two different reviewers with demonstrated high interrater reliability. Conducting focus groups with a skilled moderator using videoconferencing platforms with primary care providers is a viable option that produces sufficient levels of interaction.

## 1. Introduction

The COVID-19 pandemic disrupted face-to-face healthcare interactions and research. Healthcare providers and researchers migrated to videoconferencing environments to support social distancing and slow the spread of the virus. In-person data collection has also been impacted by the COVID-19 pandemic.

In-person interviews and focus groups, upon which qualitative research often relies, were no longer possible due to the challenges of maintaining social distancing. A focus group is an accepted qualitative research technique in which a trained moderator organizes and guides a group discussion with six to eight participants around a specific topic. The goal of a focus group is to elicit data from the group regarding the meanings, processes, and normative understanding of a topic that is deep and unlikely to be generalized to a larger population. Focus groups explicitly harness group interactions to elucidate underlying themes [1]. Research has shown focus groups to be particularly effective in exploring patients’ and providers’ opinions and experiences regarding illness and healthcare [2,3,4].

A reasonable alternative that allows for qualitative data collection to continue (despite the pandemic) is to use any of the widely available platforms used for videoconferencing. Researchers have studied the validity of online focus groups since 1997. Stewart and Williams published an analysis of the feasibility of online focus groups in 2005. They concluded that both asynchronous and synchronous focus groups could yield valuable information [5]. This was, of course, before the advent of widespread videoconferencing, which more closely mimics face-to-face interactions. Videoconferencing allows group members to see facial expressions and some body language and conveys the subtleties of tone of voice. In a study of videoconferencing for collecting qualitative data, Archibald et al. describe 16 users who cited high rates of satisfaction with the ease of use, the cost savings achieved, and the data management and security features of the platform. A total of 69% of the participants in that study preferred Zoom interviews to face-to-face meetings, phone interviews, and other videoconferencing platforms, despite the fact that 88% of the participants experienced technical difficulties [6].

Dos Santos Marques et al. describe the advantages and disadvantages of virtual focus groups [7]. Flexibility of scheduling may allow more people to participate, and participants may be more relaxed in their own homes. Researchers can also include participants who are geographically dispersed since travel is not a barrier to participation. The largest disadvantage cited was the variety of technological knowledge and expertise among participants.

Daniels et al. describe design factors to consider for focus groups using videoconferencing in healthcare [8]. These include setting explicit ground rules, offering practice sessions before focus group meetings, and providing technical support during sessions. Researchers should decide in advance how they will respond to participants who arrive late (or who leave early), those who join without video, and those who might have distractions in their environment that threaten the privacy of the focus group. With good planning and a trained moderator, Daniels et al. conclude that participant alienation can be mitigated and that there are few differences in the quality of the data collected in online, videoconferencing focus groups versus face-to-face [8].

Most qualitative researchers emphasize the content of the focus group conversation with fewer focusing on the communication processes within the group [9]. Several theoretical frameworks have been proposed to study the dynamics of the communication process. Morgan’s framework demonstrates that productive focus groups exhibit sharing and comparing. Sharing occurs when one describes one’s experiences and connects ideas to those of previous speakers. Comparing involves differentiating ideas from previous comments and expounding upon the ideas of others. Group members also show support for others by validating their contributions. In high functioning groups, members organize and conceptualize information [10]. Morgan also describes galvanizing moments in focus group interactions, i.e., ‘lightning strikes’. These occur when an idea excites the group and generates a flurry of rapid responses [9]. To capture the different types of interactions and frequencies, a modified sociogram is presented. According to Drahota and Dewey, sociograms are a useful tool to conceptualize focus group dynamics [11].

Since the COVID-19 pandemic disrupted the opportunity to conduct face-to-face focus groups for qualitative research, there have been few studies evaluating the communication processes as research transitions from face-to-face to the videoconferencing environment. This study will use Morgan’s framework to evaluate focus group interactivity following the transition to videoconferencing, using an ongoing study evaluating prescriber perceptions of deprescribing high-risk medications, as an example. The objective of this paper was to evaluate whether a focus group conducted in a videoconferencing environment can produce interaction in each of Morgan’s categories. Such evidence would support the idea that the videoconference based focus group can create an environment where participants can enter into the discussion rather than just answering the question [12].

## 2. Materials and Methods

As part of a Centers for Disease Control and Prevention (CDC)-funded, randomized controlled trial (RCT), primary care providers in two clinics in central North Carolina participated in a focus group exploring opioid and benzodiazepine prescribing and deprescribing practices for older adults via videoconferencing. The intervention included provider and patient focused education materials on deprescribing these medications, patient-specific deprescribing recommendations from a consultant pharmacist, and the support of a practice coach [13]. Focus group findings were intended to inform the content of the intervention clinics’ educational materials and provider deprescribing resources. This evaluation was conducted after the focus group transitioned to the videoconferencing environment to ensure the focus group still produced helpful content to inform the intervention.

The Institutional Review Board (IRB) of the University of North Carolina at Chapel Hill reviewed and approved this research project, IRB 18-2920. Further, the IRB approved the change from a face-to-face focus group to a videoconference-based focus group in response to the COVID-19 pandemic.

The focus group was moderated by a trained public health educator with more than fifteen years of experience in leading focus groups regarding the health needs of older adults. Focus group questions were designed by the research team comprised of faculty and staff from the University of North Carolina at Chapel Hill (UNC-CH), which included professionals from the School of Medicine, the Eshelman School of Pharmacy, the Gillings School of Global Public Health, and the Odum Institute for Research in Social Science. The interview questions underwent three rounds of revisions by this interprofessional team of experts to establish construct validity. The final version of the moderator’s guide was comprised of 20 open-ended questions, each with several corresponding probes. See Table S1.

The initial four questions explored communication with patients and asked participants to describe their experiences deprescribing opioids and benzodiazepines. Each medication class was assessed separately. Providers were also asked what advice they would give a colleague about deprescribing these medications, what prompts in the electronic medical record related to deprescribing might be helpful, what alternatives (both pharmacological and nonpharmacological) they might employ to support deprescribing, and what training they would want to better address these issues.

Originally, eight providers (three physicians, three nurse practitioners, and two physician assistants) from two clinics randomized to receive the intervention were recruited to participate in the focus group. All providers in the practices were invited to a traditional face-to-face focus group meeting that would have included dinner before the event. All eight providers agreed to join the focus group via videoconferencing when COVID-19 denied the team the face-to-face meeting. One provider did not have a webcam and was not able to complete the session because technical issues forced them to log off. Each Participant received a $100 gift card as compensation for their time.

The videoconference focus group was scheduled for a maximum of 90 min and included 20 open-ended questions. The session took place after clinic hours and was recorded using the recording feature available in the platform, Zoom Client for Meetings [computer program] Version 4.6.7. There was also a separate audiotape as a backup. The Zoom recording was professionally transcribed by an outside agency. Additionally, three note-takers were in the videoconference but were not visible to the participants, with video and audio turned off to minimize distractions.

The research team adapted many of Daniels’s recommended best practices for videoconference-based focus groups [8]. For example, ground rules and technical support were explicitly provided during the session. The first eight minutes of the session were devoted to orienting participants to the videoconferencing environment. The team also monitored for distractions in the background. There were no special technical requirements other than an internet connection, webcam, and microphone.

### Measures of Interactivity

Morgan’s model of interactivity was used to assess group dynamics and interactivity in the videoconference meeting. Two team members (CH and CK) reviewed the video recording for evidence of group members sharing, comparing, validating, organizing, conceptualizing, and producing galvanic conversation or lightning strikes [10]. Reviewers were given definitions and an example of each type of interaction. Responses were coded in only one category each. Table 1 illustrates the training of the coding process.

The reviewers tabulated the numbers of each type of response and the researchers determined the nature of the interactions following Morgan’s model. Interrater reliability was ascertained through intercorrelation coefficient calculation. Coding participant’s statements using Morgan’s categories is a qualitative process in which the researcher must catalog each comment as sharing, comparing, validating, and organizing ideas and conceptualizing them, or as a lightning strike. If there was disagreement between the reviewers, the lower number is reported along with the range. The reviewers also assessed several quantitative measures, including the number of responses made, how many participants spoke multiple times, and how often the moderator probed for additional information. Each metric was manually cataloged by each reviewer and verified by a review of the notes from the three note-takers in the session. These patterns of interactions were visualized using a modified sociogram, documenting the frequencies of the different types of interactions according to Morgan’s model.

## 3. Results

The details of the participant demographic characteristics are listed in Table 2. The participants represented 39% of all providers at these two clinics. There were four women and three men. Four and three participants were from each clinic, respectively, and were acquainted with each other before the focus group session began. Years in practice ranged from less than 5 years to more than 20 years. The focus group consisted of three physicians, two physician assistants, and two nurse practitioners.

### Focus Group Dynamics

Throughout the focus group, all seven providers maintained video and audio connection and appeared attentive and not distracted. There were no background distractions noted during the session.

The group responded to a total of 20 open-ended questions over 70 min. The moderator made use of pre-written probes for only two questions. A general open-ended probe was used to follow up on six additional questions.

An analysis of the video of the group and the notes of three independent note-takers by two reviewers (CH and CK) showed interactivity using Morgan’s measures of interactivity [10]. On average, each question generated five responses (range 2–12). The participants often responded more than once to a question (range 2–4). Multiple responses began on the first question and multiple responses increased in frequency later in the session.

The participants validated the opinions of others in at least nine statements of agreement across the 20 questions (range observed by researchers 9–12). These occurred on 10 of the 20 questions; thus, fifty percent of the questions generated statements that were encouraging and validating.

Sharing comments occurred at least seven times (25% of all questions). The reviewers noted a range of 7–11. They also observed at least seven instances in which the participants compared their idea to someone else’s in the group (range 7–10). These comparison comments increased in frequency as the group progressed.

**Figure 1:**
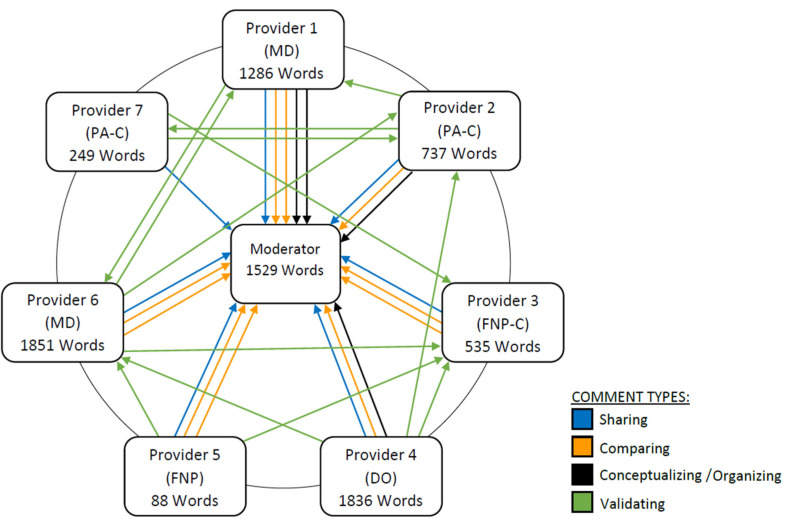
Sociogram map of interaction illustrating Morgan’s metrics of interactivity.

Organizing or conceptualizing the content of the discussion is a higher-order activity and was evidenced three or four times by observation count. The group was galvanized by a ‘lightning strike’ question toward the end of the session, which led to an extended discussion and a spontaneous case example. Both reviewers observed one lightning strike interaction. Figure 1 presents a modified sociogram showing the patterns of interaction among participants during the focus group discussion using Morgan’s metrics of interactivity. A summary of the group dynamics by the reviewers is presented in Table 3. The interrater reliability for each of Morgan’s categories assessed by the reviewers was calculated using the intraclass correlation coefficient (ICC). The ICC was 0.985 (95% CI: 0.912, 0.998), indicative of excellent interrater reliability [14].

The sociogram revealed that physicians’ word counts were higher than non-physician word counts and sometimes exceeded the word count of the moderator. All providers demonstrated sharing, comparing, and validating with two providers organizing and conceptualizing the content. Once again, these were physicians.

## 4. Discussion

Early in the COVID-19 pandemic, our research team was able to complete a focus group discussion via videoconferencing with seven primary care providers. The group demonstrated interactivity that increased over the course of the discussion. Despite the potential limitations of videoconference-based focus groups, primary care providers in our study demonstrated a depth of conversation around the topic of deprescribing opioids and benzodiazepines. Additionally, the conversation in this focus group included clear examples of interactivity in each of the categories proposed by Morgan (validation, sharing, comparing, organizing, conceptualizing, and lightning strikes), which were observed by two different reviewers with high interrater reliability [10].

All 20 questions generated responses from the participants, and more than half generated multiple responses. Only four questions (20%) did not generate discussion beyond initial responses from the group. These were questions that asked about initiating benzodiazepines. These were the same questions the group had just responded to in relation to opioids. On average, each question had five of the seven participants responding to the prompt. The final, open-ended question asked if anyone had anything else to add. No one responded to this question. In the view of the moderator and the research team, this is likely because saturation and consensus had been reached.

Half of the questions in the discussion guide produced supportive, validating statements of agreement. Repetition across questions may explain why this was not higher. In many cases, the questionnaire repeated questions exactly for two separate drug classes (opioids and benzodiazepines). Because of the repetitive nature of eight of the questions, finding evidence of Morgan’s categories of interactivity in half of the questions is actually quite high. Morgan’s categories are evidence of a productive, supportive focus group and demonstrates the participants’ willingness to discuss common struggles with deprescribing opioids and benzodiazepines.

Of note is the imbalance in participation by different members of the focus group. The sociogram revealed that three of the providers contributed significantly more content to the discussion, sometimes even exceeding the moderator in terms of word count. Upon review, it was revealed that it was the physicians who dominated the discussion. Two of the three physicians in the group were in leadership positions in their clinics and so possibly felt more of a need to address deprescribing issues or may have felt more comfortable because of their leadership role. The nurse practitioners and physician assistants had much lower word counts but all providers evidenced sharing, comparing, and validating. Many of the participants knew each other from the workplace, so interactivity may have been facilitated by these pre-existing relationships. However, we still believe that we observed a good degree of interaction, because all providers contributed to the discussion and evidenced sharing, comparing, and validating behaviors.

Qualitative research must adhere to standards of scientific rigor. Hamberg et al. describe a framework for establishing truth, consistency, neutrality, and applicability in research and map these to accepted constructs for both quantitative and qualitative research [15]. In qualitative research, these standards include credibility, dependability, confirmability, and transferability, all of which are met in our study. Credibility was established through the experience and interviewing skills of the focus group moderator and the motives of the participants to improve deprescribing practices. The independent coding of Morgan’s categories by two reviewers adds to the dependability of our findings. Neither of the two reviewers served as the focus group moderator, thus supporting the confirmability and objectivity of the findings. Finally, transferability to other settings can be interpreted through the description of the demographics and context provided for the study.

## 5. Limitations

Focus group research, including this study, is limited in that the findings cannot be generalized to larger populations. Readers may find, though, that the procedures for implementing videoconferencing focus groups are transferrable to many settings. This research is limited in that it analyzes communication processes in only one focus group that took place in central North Carolina with participants from two primary care clinics. Thus, without a comparator group, we cannot determine with certainty whether the levels of interactivity achieved in this focus group in the videoconferencing environment are comparable to what would have been achieved in person. The levels of interactions may also be influenced by the working relationships several of the providers had before the focus group was formed.

Many researchers start from the premise that face-to-face focus groups are superior to those that occur over videoconferencing. While the literature has shown some advantages of videoconferencing for focus groups in terms of recruitment, flexible scheduling, and relaxed participants, the question remains, can participants truly enter the discussion? This study showed that the same earmarks of interactivity can be achieved in focus groups in the videoconferencing environment given skillful moderating, a group that has some pre-existing relationships in the face-to-face world and a compelling topic. However, future studies are needed to directly compare the validity and value of focus groups conducted via videoconferencing versus in-person.

## 6. Conclusions

Primary care providers who participated in a focus group using a videoconferencing platform demonstrated the same types of interactivity seen in face-to-face focus groups as described by Morgan. Conducting focus group meetings with a skilled moderator using videoconferencing platforms with primary care providers is a viable option that produces sufficient levels of interaction.

## Figures and Tables

**Table 1:** Coding Process.

Morgan Category	Definition	Example
Sharing	When one describes one’s experiences and connects ideas to those of previous speakers.	“You’re right! The ones that just come in every month to have their refill at a set time. I’m also trying to have discussions of how they can try to wean their dose down.”
Comparing	Involves differentiating one’s ideas from previous comments and expounding upon the ideas of others.	“I’ve had the same experiences. I’m continuously, chronically trying to wean patients for years. Sometimes it’s been almost impossible.”
Validating	Showing support for others	“I would just like to echo what everyone has said. I don’t have anything to add, but I think those are all good points. Bravo!”
Organizing and Conceptualizing	Create a new structure or idea based on the contributions of others	“Everyone is talking about the other medicines and risks, but I think there’s a quality of life issue that you have to be interested in.”
Lightning Strikes	A galvanizing moment that creates rapid fire exchanges	“This may be way off topic but people take these medications for sleep and their sleep is not really a problem.” Followed by multiple rapid comments on sleep hygiene and medications.

**Table 2:** Characteristics of focus group participants n = 7.

Characteristic	n	Percent
**Clinical Role**		
Family Physician	3	43%
Nurse Practitioner	2	26%
Physician Assistant	2	26%
**Practice Location**		
Rural	7	100%
**Years in Practice**		
Less than 5	1	14%
5–9	1	14%
10–14	1	14%
15–19	1	14%
20–24	1	14%
Declined to answer	2	28%
**Gender**		
Female	4	57%
Male	3	43%
**Age**		
35–44	1	14%
45–54	4	57%
Declined to answer	2	28%
**Race**		
White	7	100%
**Ethnicity**		
Not Hispanic or Latino/a	7	100%

**Table 3:** Communication dynamics of video-conferenced focus group.

	n Reviewer 1	n Reviewer 2	Percentage of Questions that Evidenced the Metric
Sharing comments	7	11	25%
Comparing comments	10	7	25%
Validating comments	12	9	50%
Organizing and conceptualizing comments	4	3	5%
Lightning strikes	1	1	5%
Total responses	92	92	100%
Multiple responses by a single individual	17	17	55%
Questions moderator probed	8	8	40%

Note: comment categorization is mutually exclusive. Each comment is coded as only one type of response. Where there was disagreement between reviewers on the percentage of questions that evidenced the metric, the lower, more conservative score was used.

## Supplementary Materials

The following are available online at https://www.mdpi.com/article/10.3390/pharmacy9030117/s1. Table S1: Focus Group Questions.
