# Supplementary material for: Transitioning Focus Group Research to a Videoconferencing Environment: A Descriptive Analysis of Interactivity
Source: Pharmacy (Basel). 2021 Jun 24;9(3):117. doi: 10.3390/pharmacy9030117 (PMC8293315; doi:10.3390/pharmacy9030117)
Supplement: Supplementary file 1 [file pharmacy-09-00117-s001.zip › pharmacy-1186817-supplementary.pdf]

Table S1

**Provider Deprescribing Focus Group Questions**
**May 2020**

| **Topic and/or Question** |
| --- |
| **Q1**. When prescribing opioids for older adults (65+), what do you take into consideration?<br>• [PROBE] Patient goal(s)?<br>• [PROBE] Family goal(s)?<br>• [PROBE] Provider goal(s)?<br>• [PROBE] What indications?<br>• [PROBE] Safety vs effectiveness (risks vs benefits) |
| **Q2**. What do you tell your patients when starting an opioid for the first time?<br>• [PROBE] Appropriate use<br>• [PROBE] Length of therapy<br>• [PROBE] Safety vs effectiveness (risks vs benefits) |
| **Q3**. What do you tell your patients when refilling an opioid prescription? |
| **Q4**. Have you ever weaned a patient from an opioid, and if so, what was that experience like?<br>• [PROBE] How did you initiate that conversation with the patient?<br>• [PROBE] What, if anything, do you wish you had done differently? |
| **Q5**. When prescribing benzodiazepines for older adults (65+), what do you take into consideration?<br>• [PROBE] Patient goal(s)?<br>• [PROBE] Family goal(s)?<br>• [PROBE] Provider goal(s)?<br>• [PROBE] What indications?<br>• [PROBE] Safety vs effectiveness (risks vs benefits) |
| **Q6.** What do you tell your patients when starting a benzodiazepine for the first time?<br>• [PROBE] Appropriate use<br>• [PROBE] Length of therapy<br>• [PROBE] Safety vs effectiveness (risks vs benefits) |
| **Q7**. What do you tell your patients when refilling a benzodiazepine prescription? |
| **Q8**. Have you ever weaned a patient from a benzodiazepine, and if so, what was that experience like?<br>• [PROBE] How did you initiate that conversation with the patient?<br>• [PROBE] What, if anything, do you wish you had done differently? |
| **Q9**. What advice, if any, would you give a fellow provider about weaning a patient from opioids or benzodiazepines? |
| **Q10**. What kind of a prompt would you find useful in your electronic medical record to assist you with de-prescribing opioids and/or benzodiazepines? |
| **Q11**. Would you consider implementing this in your practice? |

| |
|---|
| • [PROBE] What are your thoughts on this guideline?<br>• [PROBE] What resources would be needed?<br>• [PROBE] What, if anything, would get in the way of implementing this? |
| **Q12**. Which pharmacological alternative therapies do you feel the most comfortable recommending for pain management? |
| **Q13.** Which pharmacological alternative therapies do you feel the most comfortable recommending for anxiety? |
| **Q14.** Which pharmacological alternative therapies do you feel the most comfortable recommending for sleep/insomnia? |
| **Q15**. Which non-pharmacological alternatives do you feel the most comfortable recommending for pain management? |
| **Q16.** Which non-pharmacological alternatives do you feel the most comfortable recommending for anxiety? |
| **Q17.** Which non-pharmacological alternatives do you feel the most comfortable recommending for sleep/insomnia? |
| **Q18**. What advice, if any, would you give a fellow provider about recommending alternatives therapies for pain management, anxiety, or sleep? |
| **Q19**. What training(s) in alternative therapies (other than medications) would be helpful for pain management, anxiety reduction, and/or sleep management? |
| **Q20**. Is there something you'd like to discuss that we haven't' talked about? |
